# Higher consumption of sugar-sweetened beverages is associated with an increased risk of endometrial cancer: a prospective cohort study of 92,777 women from the UK Biobank

**DOI:** 10.3389/fpubh.2025.1692974

**Published:** 2026-01-07

**Authors:** Ke Yi, Yuke Wu, Dan Liu, Kunyan Zhou

**Affiliations:** 1 Department of Obstetrics and Gynecology, West China Second University Hospital, Sichuan University, Chengdu, China; 2 Key Laboratory of Birth Defects and Related Diseases of Women and Children (Sichuan University), Ministry of Education, Chengdu, China; 3 Department of Ultrasonic Medicine, West China Second University Hospital of Sichuan University, Chengdu, China

**Keywords:** dietary sugars, fruit and vegetable juices, non-nutritive sweeteners, prospective studies, uterine neoplasms

## Abstract

**Introduction:**

The association between three beverage types—sugar-sweetened, artificially sweetened, and natural juices—and the incidence of endometrial cancer (EC) in a large cohort of women, given that this association is not yet fully understood.

**Methods:**

We conducted a prospective, population-based cohort study using data from 92,777 women in the UK Biobank with a median follow-up of 13.3 years. Cox proportional hazards regression and substitution analyses were used to evaluate the associations.

**Results:**

During follow-up, 682 new cases of EC were recorded. Higher consumption of sugar-sweetened beverages (SSBs) was significantly associated with an increased EC risk (HR 1.29 for >1 unit/day vs. non-consumers, P for trend = 0.004), while no statistically significant associations were found for artificially sweetened beverages (ASBs) or natural juices. Replacing SSBs with ASBs or natural juices was associated with a 12 and 9% reduction in EC risk, respectively, and body mass index (BMI) was found to mediate 18.2% of the association.

**Conclusion:**

In conclusion, higher consumption of SSBs is linked to an increased risk of EC, and substituting these drinks may help reduce this risk, highlighting the role of dietary choices in EC prevention.

## 1 Introduction

Endometrial cancer (EC) ranks among the most common gynecologic malignancies, and has a consistently increasing incidence and associated mortality on a global scale (1). As a result, EC presents as an escalating public health challenge (1). In 2020, the global incidence of EC was estimated at 417,336 new cases (2), with the majority of diagnoses made in postmenopausal women characterized by a peak incidence typically between 65 and 75 years of age (3). To date, there is convincing evidence that indicates age, genetic predisposition, family history, and endogenous or exogenous estrogen exposure are common risk factors for EC, and also that obesity, the metabolic syndrome, and diabetes are well-established diet-related risk factors associated with about 70% of EC cases (4). Diet may affect EC risk either directly or indirectly through its impact on body weight and metabolic health, although the underlying mechanisms remain poorly understood (5). As a modifiable factor, diet plays a crucial role in preventing EC (6) and therefore early dietary intervention should be actively promoted. Natural compounds play multiple vital roles in the prevention and treatment of chronic diseases, including antioxidant, anti-inflammatory, and blood glucose and lipid regulation effects, offering the advantage of being free from side effects (7–10).

Beverage consumption is a key component of the diet. Sugars in beverages are metabolized and absorbed more rapidly than those in whole foods (11). Beverages, being a major source of free sugars have been linked to different metabolic health effects (12). There is now considerable evidence that metabolic disorders are an established risk factor for EC (4). On this basis, we hypothesized that intake of sugar-sweetened beverages (SSBs) is associated with an increased risk of EC. However, evidence supporting this association remains limited and the potential role of these beverages in EC development has been largely overlooked. Growing evidence indicates that high dietary sugar intake is detrimental to health, particularly regarding cancer risk and all-cause mortality (5, 13, 14). A recent meta-analysis (15) showed that the consumption of SSBs was associated positively with the risk of several cancers, including breast, colorectal, biliary tract, and liver cancer. In response to this concern, the Dietary Guidelines for Americans (2020–2025) and Health Canada recommend limiting added sugar intake to less than 10% of total daily energy intake, while the UK National Health Service advises that the intake should be restricted further to below 5% of overall energy intake (16–18). It is worth noting that, although beverages containing artificial sweeteners are often viewed as substitutes for those with added sugars, a growing body of evidence suggests these sweeteners could be associated with adverse health effects, including increased risks of type 2 diabetes, obesity, and all-cause mortality (19–21). Nevertheless, the relationship between consumption of artificially sweetened beverages (ASBs) and cancer incidence is still debated. Evidence from a large-scale French cohort indicated that higher total intake of artificial sweeteners was linked to a 15% greater overall cancer risk and a 13% higher risk for obesity-related cancers, relative to non-consumers (5). A recent meta-analysis further reported that greater intake of ASBs was associated with a higher likelihood of developing leukemia, but was inversely related to colorectal cancer incidence (22). Consistent with these results, the American Diabetes Association recommend cautious use of artificial sweeteners and suggested that while they may serve as temporary substitutes for added sugars, they should not be relied upon as a long-term strategy for weight management or disease prevention (23). Even natural juices, despite containing beneficial nutrients, are high in naturally occurring fructose, which should be consumed with caution as its intake has been linked to metabolic disorders, including obesity, metabolic syndrome, and type 2 diabetes mellitus (24). Nonetheless, the relationship between ASBs consumption and cancer occurrence is still debated. Supporting this uncertainty, a recent meta-analysis suggested that greater intake of fruit juice could be associated with an elevated cancer risk (22), whereas a U.S. cohort study reported that fruit juice had no significant association with liver cancer (15).

The potential role of consuming the three main types of beverages in EC prevention has been largely overlooked in current clinical recommendations, despite growing evidence linking these beverages to EC-related risk factors (25). The link between consuming sweetened beverages and the risk of EC is still not well established and has shown mixed results (26–28). For instance, findings from the Swedish Mammography Cohort Study showed no significant difference in EC risk between SSBs consumers and non-consumers (28). In contrast, findings from the U.S.-based Iowa Women’s Health Study indicated that women consuming any quantity of SSBs had a markedly elevated risk of EC relative to non-consumers. Conversely, another investigation found no significant association between EC risk and intake of ASBs or fruit juice (27). However, evidence from a recent Canadian cohort indicated that greater intake of SSBs and fruit juice correlated with a higher incidence of EC (26). Given these inconsistent findings, we conducted a prospective cohort study using the UK Biobank to examine the relationship between the consumption of three beverage types—sugar-sweetened, artificially sweetened, and natural juices—and EC risk. Additionally, we assessed how substituting one beverage for another might impact EC risk, leveraging the large sample size and extensive follow-up of the UK Biobank cohort.

## 2 Methods

### 2.1 Study population

This research utilized data from the UK Biobank, a large-scale, population-based prospective cohort comprising more than 500,000 individuals aged 40–69 years, recruited between 2006 and 2010 from 22 assessment centers located in England, Wales, and Scotland. At baseline, the participants were required to complete a touch-screen questionnaire addressing their lifestyle, dietary habits, physical activity, medical history, and other prior exposures. The participants also provided biological samples (blood and urine) for a range of assays. The detailed protocol of UK Biobank can be accessed at www.ukbiobank.ac.uk (29). Written informed consent in electronic form was provided by all participants at enrolment. The research was conducted in accordance with the Strengthening the Reporting of Observational Studies in Epidemiology (STROBE) guidelines.

**Figure 1:**
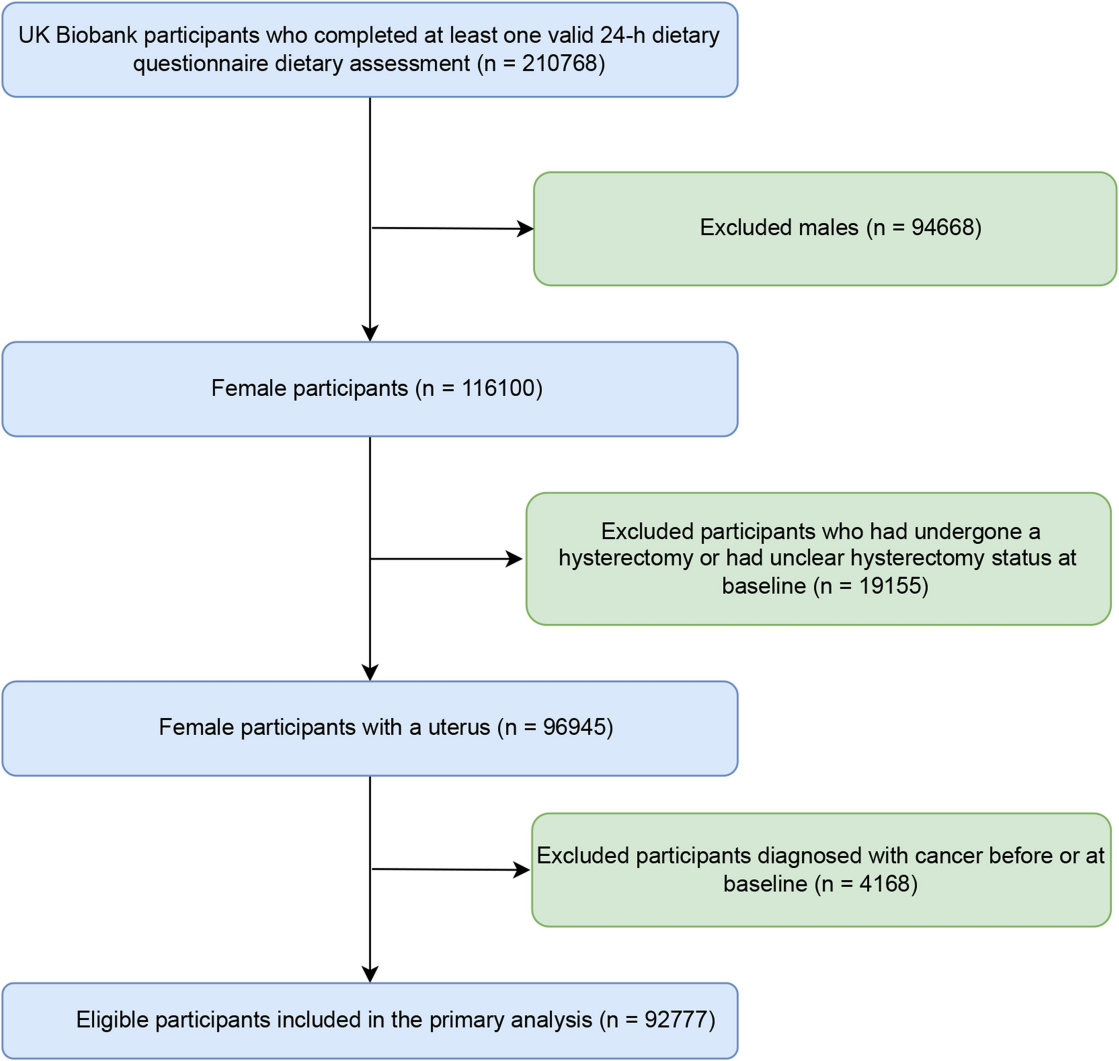
Flowchart of participant selection and inclusion.

The current study enrolled 210,768 participants who had completed more than one online dietary questionnaire for the UK Biobank between 2006 and 2010. We subsequently excluded male participants (*n* = 94,668), females who had undergone a hysterectomy or had an indeterminate hysterectomy status at baseline (*n* = 19,155), and individuals diagnosed with cancer prior to or at baseline (*n* = 4,168). As shown in Figure 1, the final analytical cohort included 92,777 participants. The UK Biobank received ethical approval from the North West-Haydock Research Ethics Committee (REC reference: 16/NW/0274). All participants in this study provided informed consent when they were recruited.

### 2.2 Exposure assessment

The intake of beverages was collected utilizing Oxford Web-Q, an online 24-h dietary recall questionnaire administered via a touch-screen interface. Data collection occurred annually from 2009 to 2012, with up to five collections per participant. The participants reported their consumption of beverages from the previous day, specifying the number (0, 0.5, 1, 2, 3, 4, 5, or more than 6) of units consumed (cans/glasses/250 mL cartons). In the present study, SSBs were categorized as fizzy drinks (field id 100,170) and squash (A beverage produced by removing a certain percentage of natural water content from pure fruit juice using specific methods, field id 100,180), while ASBs were identified as low-calorie drinks (field id 100,160). In accordance with previous research (5, 30, 31) natural juices were defined as pure orange juice (field id 100,191), grapefruit juice (field id 100,200), and other pure fruit or vegetable juices (field id 100,210). The mean beverage consumption from the five collections was classified into three intake categories: non-consumers (0 unit/day), moderate consumers (> 0–1 unit/day), and high consumers (> 1 unit/day) which were then used to estimate the usual consumption as exposure.

### 2.3 Outcome assessment

The primary endpoint was newly diagnosed EC identified during follow-up. Cancer cases were coded according to the International Classification of Diseases, 10th Revision (ICD-10), with EC defined by code C54.1. Data on incident cancer cases were sourced from national cancer registries, which consolidated information from various regional cancer centers across the United Kingdom. Each participant was followed from baseline until the earliest of the following events: occurrence of the outcome, death, loss to follow-up, or the end of the follow-up period. The end of follow-up was determined by the most recent date of available health records, which was October 31, 2022 for England, July 31, 2021 for Scotland, and February 28, 2018 for Wales.

### 2.4 Covariate assessment

Based on earlier research (31, 32) the covariates including sociodemographic, lifestyle, comorbidities, and female reproductive factors were chosen to be potential confounders. Sociodemographic factors included age, education level (unknown, college, or other levels), gender (male or female), and household income (categorized as < £18,000, £18,000-30,999, £31,000-51,999, £52,000-100,000, or > £100,000). Socioeconomic status was assessed using the Townsend Deprivation Index (TDI). Ethnicity was self-reported and classified as White or non-White (including Asian, Black, multiethnic, and other groups). Lifestyle factors included smoking statusand drinking alcohol status (never, previous, or current). Physical activity status (yes or no) was determined using the International Physical Activity Questionnaire (IPAQ). Participants were classified as active if they achieved ≥150 min/week of moderate activity, ≥75 min/week of vigorous activity, or an equivalent combination of both (33). Female reproductive factors included age at menarche, number of live births, use of hormone replacement therapy (HRT) (yes or no), use of oral contraceptives (OCs) (yes or no), and menopausal status (yes, no, or unsure). Body mass index (BMI), calculated as weight (kg)/height^2^ (m^2^), and classified according to WHO criteria as underweight (<18.5 kg/m^2^), normal (18.5–24.9 kg/m^2^), overweight (25.0–29.9 kg/m^2^), or obese (≥30 kg/m^2^) (34). Comorbidity variables included hypertension defined as a systolic measurement of ≥ 130 mmHg and/or a diastolic measurement of ≥ 85 mmHg, or the current use of antihypertensive medication (35); and diabetes mellitus identified based on a fasting blood glucose level of ≥ 11.1 mmol/L, hemoglobin A1c (HbA1c) level of ≥ 6.5% (48 mmol/mol), self-reported history of diabetes prior to baseline, or the use of glucose-lowering medication (36). The methodology for data collection has been detailed in a previous publication (37). All field identifiers used in the study are shown in Supplementary Table S1.

For the 29,027 missing data points, continuous variables were imputed with their median, while categorical variables were replaced with the mode, defined as the most frequently occurring category. Specifically, missing data were observed for ethnicity (*n* = 211, 0.23%), TDI (*n* = 114, 0.12%), BMI (*n* = 225, 0.24%), smoking status (*n* = 179, 0.19%), drinking status (*n* = 53, 0.06%), physical activity (*n* = 875, 0.94%), household income (*n* = 11,136, 12.0%), diabetes mellitus (*n* = 17,364, 18.7%), menarche age (*n* = 2,529, 2.73%), live birth number (*n* = 42, 0.05%), HRT use (*n* = 195, 0.21%), OCs use (*n* = 138, 0.15%), and menopause status (*n* = 79, 0.09%). All field IDs are provided in Supplementary Table S1.

### 2.5 Statistical analysis

The baseline characteristics of the participants with different options for SSBs were analysed. Continuous data were summarized as mean ± standard deviation (SD) for normally distributed variables and as median with interquartile range (IQR) for skewed data. Categorical data were presented as frequencies and percentages. Cox proportional hazards regression was applied to evaluate associations between the three beverage types and EC incidence, reporting hazard ratios (HRs) with 95% confidence intervals (CIs). The crude model included no covariates, Model 1 was adjusted for age, ethnicity, drinking status, smoking status, TDI, BMI, and education, while Model 2 was adjusted additionally for live birth number, hypertension, diabetes mellitus, menarche age, menopause status, HRT use, OCs use, and physical activity. The proportional hazards (PH) assumption for all fully adjusted Cox models was evaluated using Schoenfeld residuals, and no violations were detected for the beverage exposure variables (Supplementary Table S2). Cumulative incidence and survival curves for EC were generated by beverage type, using a Cox proportional hazards model fully adjusted for Model 2 covariates.

Non-linear associations between beverage intake and EC risk were examined using restricted cubic splines (RCS) with four knots placed at the 5th, 35th, 65th, and 95th percentiles of intake, fully adjusted for Model 2 covariates. In addition, one-to-one (isovolumetric) substitution analyses were performed to estimate the effect on EC risk of replacing one beverage type with an equivalent unit of another while keeping the total beverage intake (unit/day) constant (38). Mediation analysis was performed to quantify the proportion of the association between SSBs intake and EC risk attributable to BMI (indirect/total effect). Several sensitivity analyses and subgroup analyses were undertaken to assess the consistency of our findings. (1) We repeated the primary analysis omitting participants with incomplete covariate data. (2) To reduce measurement error and improve the accuracy of the estimation of habitual intake we limited the analysis to individuals who had completed more than two repeated dietary assessments. (3) For consistency and to avoid potential time-related biases, the dietary data used were those from the first finished questionnaire rather than the mean of the multiple assessments. (4) To minimize the potential for reverse causation, participants diagnosed with EC within the first three years of follow-up were excluded from the analysis. (5) We adjusted for household income in place of the TDI.

The statistical analyses were performed using the UK Biobank Research Analysis Platform with R software (version 4.4.1) and FaskUKB packsges (39). A two-sided *p*-value < 0.05 indicated statistical significance.

## 3 Results

### 3.1 Baseline characteristics of the participants

Table 1 presents baseline characteristics stratified by SSBs intake. The analysis included 92,777 participants, with a mean age of 55.0 years (SD 8.0) and 95% identifying as white. Among them, 28,746 (30.9%) reported SSBs consumption, 19,947 (21.5%) consumed ASBs, and 46,190 (49.8%) consumed natural juices. Individuals with higher SSBs intake (>1 unit/day) had greater mean BMI values and higher prevalence of overweight (34%) and obesity (23%). They were also more likely to report hormone replacement therapy (HRT) use (25%) than non-consumers. Over a median follow-up of 13.3 years (IQR 12.7–14.1), 682 incident EC cases (0.74%) were recorded.

**Table 1:** Baseline characteristics of participants grouped according to their consumption of SSBs.

Characteristic	SSBs (units/day)			
	Overall (*n* = 92,777)	0 (*n* = 64,531)	> 0–1 (*n* = 19,356)	> 1 (*n* = 8,890)
Age, year	55.0 ± 8.0	55.0 ± 8.0	55.0 ± 8.0	53.0 ± 8.0
TDI	−1.52 ± 2.86	−1.53 ± 2.87	−1.60 ± 2.82	−1.35 ± 2.93
BMI, kg/m^2^	26.3 ± 5.0	26.2 ± 4.9	26.3 ± 4.9	27.0 ± 5.5
Obesity (%)				
Underweight	819 (0.9%)	602 (0.9%)	155 (0.8%)	62 (0.7%)
Normal	42,424 (46%)	29,913 (46%)	8,779 (45%)	3,732 (42%)
Obese	17,442 (19%)	11,729 (18%)	3,636 (19%)	2,077 (23%)
Overweight	31,867 (34%)	22,125 (34%)	6,742 (35%)	3,000 (34%)
Education (%)				
Unknown	7,044 (7.6%)	5,085 (7.9%)	1,365 (7.1%)	594 (6.7%)
College	40,388 (44%)	28,382 (44%)	8,429 (44%)	3,577 (40%)
Other levels	45,345 (49%)	31,064 (48%)	9,562 (49%)	4,719 (53%)
Ethnicity (%)				
Non-white	4,219 (4.6%)	2,718 (4.2%)	983 (5.1%)	518 (5.8%)
White	88,347 (95%)	61,675 (96%)	18,321 (95%)	8,351 (94%)
Alcohol drinking status (%)				
Never	3,731 (4.0%)	2,425 (3.8%)	856 (4.4%)	450 (5.1%)
Previous	2,679 (2.9%)	1,765 (2.7%)	565 (2.9%)	349 (3.9%)
Current	86,314 (93%)	60,305 (94%)	17,920 (93%)	8,089 (91%)
Smoking status (%)				
Never	56,913 (61%)	39,059 (61%)	12,339 (64%)	5,515 (62%)
Previous	29,433 (32%)	20,970 (33%)	5,785 (30%)	2,678 (30%)
Current	6,252 (6.8%)	4,384 (6.8%)	1,192 (6.2%)	676 (7.6%)
Physical activity (%)	55,660 (61%)	39,058 (61%)	11,385 (59%)	5,217 (59%)
Household income, £/year (%)				
< 18,000	12,910 (16%)	8,956 (16%)	2,636 (16%)	1,318 (17%)
18,000 to 30,999	19,961 (24%)	13,787 (24%)	4,219 (25%)	1,955 (25%)
31,000 to 51,999	23,158 (28%)	16,046 (28%)	4,847 (29%)	2,265 (29%)
52,000–100,000	19,792 (24%)	13,770 (24%)	4,171 (25%)	1,851 (23%)
> 100,000	5,820 (7.1%)	4,211 (7.4%)	1,114 (6.6%)	495 (6.3%)
Menarche age (%)	13.0 ± 2.0	13.0 ± 2.0	13.0 ± 2.0	13.0 ± 2.0
Live birth number (%)				
0	21,378 (29%)	14,664 (29%)	4,420 (29%)	2,294 (32%)
1	12,463 (17%)	8,628 (17%)	2,527 (16%)	1,308 (18%)
2	39,832 (54%)	27,762 (54%)	8,503 (55%)	3,567 (50%)
HRT use (%)	26,836 (29%)	19,012 (30%)	5,606 (29%)	2,218 (25%)
OCs use (%)	79,114 (85%)	54,933 (85%)	16,485 (85%)	7,696 (87%)
Menopause status (%)				
No	29,514 (32%)	19,564 (30%)	6,290 (33%)	3,660 (41%)
Not sure	5,071 (5.5%)	3,406 (5.3%)	1,058 (5.5%)	607 (6.8%)
Yes	58,113 (63%)	41,516 (64%)	11,986 (62%)	4,611 (52%)
Hypertension (%)	53,956 (58%)	37,686 (58%)	11,229 (58%)	5,041 (57%)
Diabetes mellitus (%)	2,616 (3.5%)	1,923 (3.7%)	472 (3.0%)	221 (3.1%)

Continues variables are displayed as mean ± SD and categorical variables as numbers (%).

BMI, body mass index; TDI, Townsend Deprivation Index; SD, standard deviation; HRT, hormone replacement therapy; OCs, oral contraceptives; SSBs, Sugar-sweetened beverages.

### 3.2 Beverage type intake and its association with endometrial cancer risk

**Figure 2:**
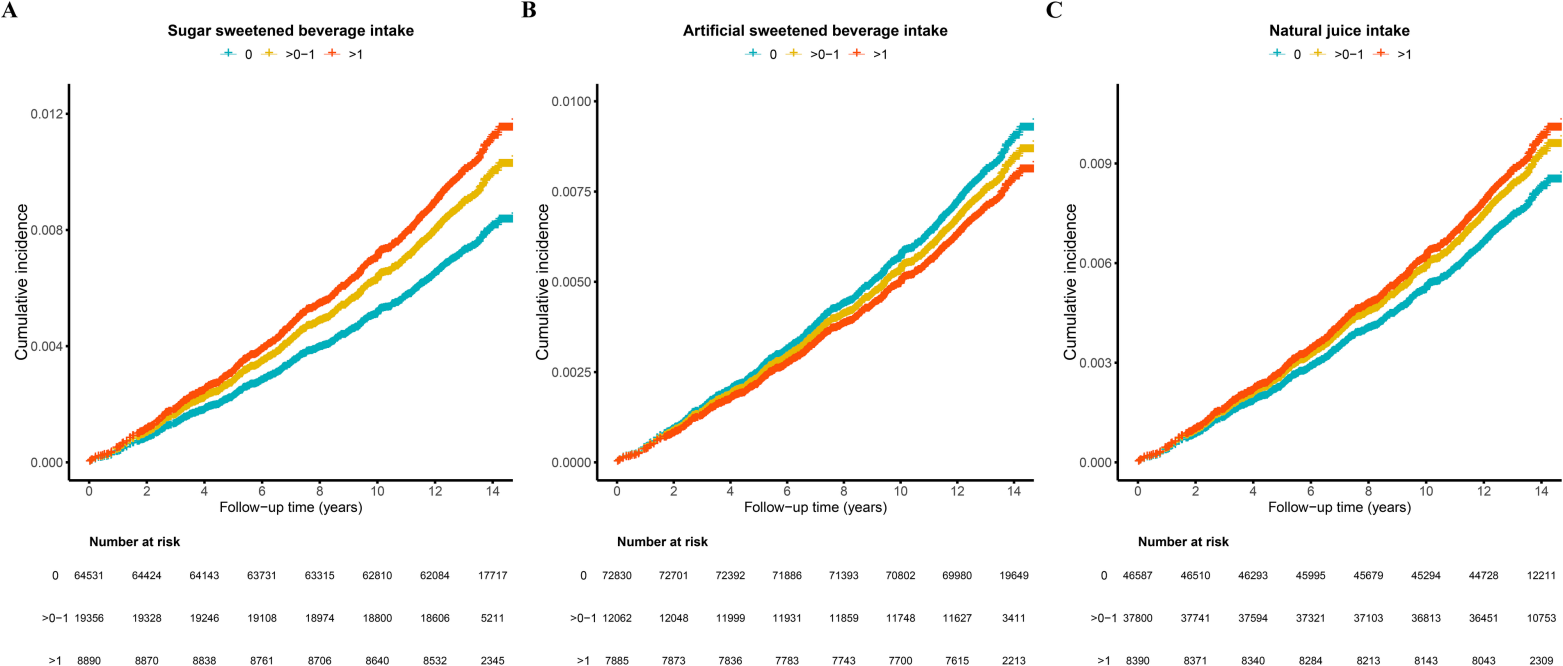
Survival analysis assessment of endometrial cancer incidence in relationship to beverage consumption (unit/day). **(A)** Sugar sweetened beverage intake; **(B)** ASBs intake; **(C)** Natural juice intake. The curves are adjusted for age, ethnicity, drinking status, smoking status, TDI, BMI, education, live birth number, hypertension, diabetes mellitus, menarche age, menopause status, HRT use, OCs use, and physical activity. BMI, body mass index; TDI, Townsend Deprivation Index; HRT, hormone replacement therapy; OCs, oral contraceptives; ASBs, Artificial sweetened beverage.

There was a notable positive link between an increased intake of sugar-sweetened drinks and the risk of EC. A positive but non-significant association was observed for natural juices, whereas an inverse but non-significant association was observed for intake of ASBs (Table 2; Figure 2).

**Table 2:** Risk of endometrial cancer associated with intake of the three types of beverages.

Beverages	Case/events	Crude HR (95%CI)	*p*	Model 1 HR (95%CI)	*p*	Model 2 HR (95%CI)	*p*
SSBs (unit/day)							
0	439/64092	1.00 (ref)		1.00 (ref)		1.00 (ref)	
> 0–1	167/19189	1.27 (1.06–1.52)	0.008	1.28 (1.07–1.53)	0.007	1.28 (1.07–1.53)	0.007
> 1	76/8814	1.26 (0.99–1.61)	0.061	1.3 (1.02–1.66)	0.037	1.29 (1.01–1.65)	0.040
P for trend			0.007		0.003		0.004
ASBs (unit/day)							
0	539/72291	1.00 (ref)		1.00 (ref)		1.00 (ref)	
> 0–1	88/11974	0.98 (0.78–1.23)	0.875	0.9 (0.72–1.13)	0.364	0.9 (0.71–1.13)	0.354
> 1	55/7830	0.94 (0.71–1.24)	0.668	0.8 (0.6–1.07)	0.129	0.79 (0.6–1.06)	0.113
P for trend			0.664		0.095		0.083
Natural juices (unit/day)							
0	326/46261	1.00 (ref)		1.00 (ref)		1.00 (ref)	
> 0–1	285/37515	1.07 (0.91–1.25)	0.401	1.11 (0.95–1.3)	0.203	1.11 (0.94–1.3)	0.211
> 1	71/8319	1.21 (0.93–1.56)	0.150	1.26 (0.97–1.63)	0.082	1.25 (0.97–1.62)	0.085
P for trend			0.142		0.057		0.060

Crude model adjusted for no covariates.

Model 1 adjusted for age, ethnicity, drinking status, smoking status, TDI, BMI, and education.

Model 2 adjusted for age, ethnicity, drinking status, smoking status, TDI, BMI, education, live birth number, hypertension, diabetes mellitus, menarche age, menopause status, HRT use, OCs use, and physical activity.

BMI, body mass index; TDI, Townsend Deprivation Index; HRT, hormone replacement therapy; OCs, oral contraceptives; HR, hazard ratio; CI, confidence interval; ref, reference, SSBs, Sugar-sweetened beverages; ASBs, Artificial sweetened beverage.

In the fully adjusted model 2, participants consuming 0–1 unit/day of SSBs had a HR of 1.28 (95% CI, 1.07–1.53; *p* = 0.007), whereas those consuming more than 1 unit/day had an HR of 1.29 (95% CI, 1.01–1.65; *p* = 0.04), compared with that of non-consumers. A significant dose–response relationship was identified between SSBs consumption and the risk of EC (P for trend = 0.004) (Table 2). Furthermore, the survival curve adjusted for all the covariates showed an increased risk of incident EC among participants who consumed more than 1 unit/day of SSBs (Figure 2).

In the fully adjusted model 2, compared with participants consuming 0 unit/day of ASBs, those consuming 0 to 1 unit per day had a HR of 0.90 (95% CI, 0.71–1.13; *p* = 0.354), while those consuming > 1 unit/day had an HR of 0.79 (95% CI, 0.60–1.06; *p* = 0.113). Although a dose–response trend was observed, the association did not reach statistical significance (P for trend = 0.083) (Table 2). The adjusted survival analysis, demonstrated that participants who consumed > 1 unit/day of ASBs had a lower incidence of EC compared to that of non-consumers (Figure 2).

In the fully adjusted model 2, participants consuming 0–1 unit/day of natural juice had a HR of 1.11(95% CI, 0.94–1.3; *p* = 0.211), while those consuming more than 1 unit/day had an HR of 1.25 (95% CI, 0.97–1.62; *p* = 0.085) compared with that of non-consumers. A dose–response relationship was observed between consumption of natural juices and the risk of EC (P for trend = 0.06) (Table 2). After adjustment for all the covariates, the survival analysis also demonstrated that participants who consumed more than 1 unit/day of natural juice compared to that of non-consumers had a higher risk of incident EC (Figure 2).

**Figure 3:**
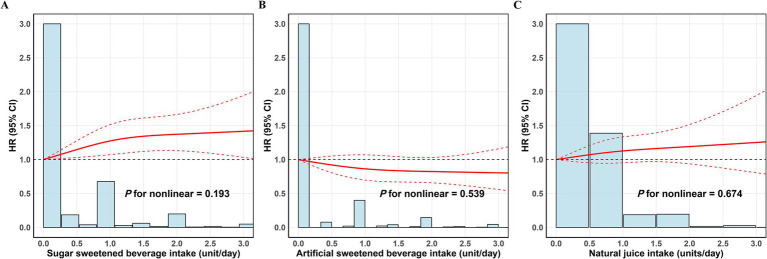
Nonlinear association between beverage intake and the risk of endometrial cancer. **(A)** Sugar sweetened beverage intake; **(B)** ASBs intake; **(C)** Natural juice intake. Adjusted for age, ethnicity, drinking status, smoking status, TDI, BMI, education, live birth number, hypertension, diabetes mellitus, menarche age, menopause status, HRT use, OCs use, and physical activity. BMI, body mass index; TDI, Townsend Deprivation Index; HRT, hormone replacement therapy; OCs, oral contraceptives; HR, hazard ratio; CI, confidence interval; ASBs, Artificial sweetened beverage.

RCS analysis revealed no evidence of a non-linear association between intake of the three beverage types and EC incidence. Nonetheless, higher consumption of SSBs (P for nonlinear = 0.193) and natural juice was linked to elevated EC risk ((P for nonlinear = 0.674)), while greater intake of ASBs correlated with reduced risk (P for nonlinear = 0.539). These results were consistent with those from the primary analyses (Figure 3).

### 3.3 Substitution analyses

Replacing one unit/day of SSBs with ASBs, natural juice, or water was associated with 12% (95% CI, 0.81–0.96; *p* = 0.004), 9% (95% CI, 0.83–0.99; *p* = 0.042), and 5% (95% CI, 0.90–1.01; *p* = 0.059) lower EC risk, respectively. Conversely, replacing one unit/day of ASBs or natural juice with SSBs corresponded to a 13% (95% CI, 1.04–1.24; *p* = 0.004) or 10% (95% CI, 1.01–1.20; *p* = 0.042) higher risk, respectively. No significant risk differences were observed when water or ASBs were used as replacements (Table 3).

**Table 3:** Substitution analysis examining the association between the risk of endometrial cancer and beverage intake.

Substitution analysis	SSBs HR (95%CI)	*p*	ASBs HR (95%CI)	*p*	Natural juices HR (95%CI)	*p*	Water intake HR (95%CI)	*p*
With SSBs	ref	NA	1.13 (1.04–1.24)	0.004	1.1 (1.01–1.2)	0.042	1.05 (1.01–1.11)	0.059
With ASBs	0.88 (0.81–0.96)	0.004	ref	NA	0.95 (0.87–1.04)	0.24	1 (0.94–1.05)	0.883
With natural juices	0.91 (0.83–0.99)	0.042	1.05 (0.97–1.15)	0.24	ref	NA	1.02 (0.96–1.08)	0.582
With water intake	0.95 (0.9–1.01)	0.059	1 (0.95–1.06)	0.883	0.98 (0.93–1.04)	0.582	ref	NA

HR, hazard ratio; CI, confidence interval; NA, not applicable, SSBs, Sugar-sweetened beverages; ASBs, Artificial sweetened beverage.

### 3.4 Mediation analysis

In the mediation analysis, BMI accounted for part of the effect between SSBs consumption and the risk of EC, with an estimated HR of 1.038 (95% CI, 1.025–1.053) and a mediation proportion of 18.2% (Supplementary Table S3).

### 3.5 Subgroup and sensitivity analysis

Subgroup analyses revealed no significant interactions between beverage intake and EC risk across predefined strata, including obesity, smoking, alcohol consumption, hypertension, diabetes, HRT use, and OC use (P for interaction > 0.10; Supplementary Table S4).

Sensitivity analyses yielded consistent results. The association between SSBs intake and EC risk remained positive across all models, though not always statistically significant, with effect sizes largely stable. ASBs were consistently linked to lower EC risk, whereas natural juice intake was associated with higher risk. Specifically: (1) Excluding participants with missing covariates showed a positive but non-significant trend for >1 unit/day of SSBs (HR = 1.24, 95% CI: 0.89–1.72; *p* = 0.197; Supplementary Table S5); (2) restricting to those with >2 dietary assessments yielded a significant positive association (HR = 1.38, 95% CI: 1.01–1.88; *p* = 0.045; Supplementary Table S6); (3) using only the first dietary questionnaire maintained a positive but non-significant association (HR = 1.39, 95% CI: 0.82–2.36; *p* = 0.218; Supplementary Table S7); (4) excluding EC cases within the first 3 years strengthened the significance (HR = 1.41, 95% CI: 1.06–1.87; *p* = 0.018; Supplementary Table S8); and (5) replacing TDI with household income produced similar results (HR = 1.29, 95% CI: 1.01–1.66; *p* = 0.04; Supplementary Table S9).

## 4 Discussion

### 4.1 Summary of findings

In this large prospective cohort of 92,777 UK Biobank women, intake of SSBs exceeding 1 unit/day was associated with higher EC risk compared with non-consumers (*p* = 0.040), whereas no significant associations were observed for ASBs or natural juices (*p* > 0.05). These results remained robust across multiple sensitivity analyses. Substituting one unit/day of SSBs with ASBs or natural juice was linked to reduced EC risk, while reverse substitutions were associated with increased risk. Overall, these findings add to the evidence supporting beverage intake as a modifiable factor in EC prevention and underscore the potential benefit of dietary modification.

### 4.2 Comparison with previous studies

To the best of our knowledge, evidence on the association between sweetened beverages and the incidence risk of EC was relatively limited and inconclusive, with prior studies reporting inconsistent findings (26–28). A U.S.-based cohort consisting of 23,039 postmenopausal women, reported a statistically significant 78% increased risk of EC in participants who consumed SSBs compared to non-consumers (27). Our study showed a similar positive association, although the magnitude of risk was considerably lower, with only a 28% increased risk with consumption of SSB compared to non-consumption.

Several factors may explain this discrepancy. First, our study population included both premenopausal and postmenopausal women, whereas the U.S. study was restricted to postmenopausal women, a group known to have a higher baseline risk of EC (3). Second, the proportion of individuals consuming SSBs was substantially higher in the U.S. cohort (56%) compared to our UK-based population (40%). This finding indicated different consumption patterns may partly explain the variation in risk estimates. More recently, a Canadian cohort study suggested that high intake of SSBs was associated with a 58% increased risk of developing EC (26), a rate consistent with that of our study. In contrast to our findings, the Swedish Mammography Cohort, which included 61,226 women, reported no significant difference in EC risk between consumers and non-consumers of SSBs (28). This Swedish study obtained dietary data through the administration of food frequency questionnaires (FFQs) at two time points, initially in 1990 and subsequently in 1997. The initial FFQ evaluated the frequency of consumption for 67 food items over the preceding 6 months, whereas the follow-up FFQ expanded its scope to include 96 food items and assessed dietary intake over the preceding twelve months. The wide recall periods and differences in questionnaire content may have introduced considerable recall bias. Moreover, discrepancies between the two FFQs, particularly regarding the categorization and reporting of SSBs, fruit juice, and ASBs, may have led to measurement errors in data merging. These methodological differences may partially explain the inconsistency between the findings of the two studies.

In the UK dietary context, one beverage unit in the Oxford WebQ corresponds to approximately 250 mL. Based on the UK McCance & Widdowson food composition tables, most sugar-sweetened beverages contain 8–10 g of sugar per 100 mL; thus, one unit typically provides about 20–25 g of free sugars. This amount is sufficient to generate a substantial glycemic and insulinemic response (16, 40). SSBs are the principal provider of dietary free sugars and are known to induce a high glycemic load, resulting in an acute increase in postprandial blood glucose levels and stimulation of insulin secretion. A high glycemic load has been linked to insulin resistance, systemic inflammation, and visceral fat accumulation, metabolic disturbances that are well-established risk factors for obesity and type 2 diabetes (41). These metabolic abnormalities are also established risk factors for EC, particularly in postmenopausal women, due to increased peripheral aromatization of androgens to estrogens in adipose tissue, which increases endogenous estrogen levels in the absence of ovarian estrogen production (42). Our study showed that BMI explained part of the effect between SSBs consumption and the developing risk of EC. This finding indicates adiposity may be a potential biological pathway involved in the development of the malignancy. The detailed results of this mediation analysis are presented in Supplementary Table S1. This finding aligns with previous studies and adds strength to these findings by quantifying the indirect effect through BMI (43). Given the limitations and inconsistent findings of previous studies, our results provide further evidence to support public health recommendations to limit SSBs consumption as a potential strategy for EC prevention, especially among individuals at higher metabolic risk.

Previous studies have suggested that replacing sugar with ASBs may lower caloric intake and glycemic load (44). Nevertheless, the impact of these factors on obesity, diabetes, and cancer continues to be a subject of debate within the academic community. Our study showed the association between intake of ASBs and EC risk was not statistically significant, although a positive trend was observed that suggested further investigation into potential underlying mechanisms is warranted. This finding of a lack of association aligns with the results of the previous cohort study in the U.S., which also showed no significant association between consumption of ASBs and the risk of EC (27).

Notably, replacing one unit/day of SSBs with ASBs was linked to a 12% lower risk of EC, underscoring the potential advantage of substitution strategies over total intake reduction. It should be noted that UK Biobank does not provide brand-specific sweetener information. In the UK, most low-calorie beverages typically contain aspartame, acesulfame-K, or sucralose, which should be considered when interpreting the substitution effect. However, despite the observed potential benefit, the long-term health effects of ASBs remain controversial. Some studies suggest they may reduce calorie intake and lower the risk of obesity and diabetes, whereas others raise concerns about potential metabolic consequences (45), concerns persist regarding their broader health implications. However, evidence from prospective cohort studies has shown that higher intake of both sugar or ASBs are linked to greater accumulation of visceral adipose tissue (46). Similarly, animal studies have reported no significant differences between SSBs and ASBs in terms of retroperitoneal fat mass or changes in body weight (42). Therefore, although our findings suggest that ASBs may act as a beneficial replacement to SSBs for reducing EC risk, these results should be interpreted with caution. Further research is needed to confirm these findings and clarify the biological mechanisms linking beverage consumption to EC risk.

The current study showed a non-significant positive trend between higher natural juice consumption and EC risk. Notably, substituting 1 unit/day of SSBs with natural juices was associated with a 9% reduction in EC risk, suggesting that replacement strategies may confer greater benefit than absolute intake reduction. This potential protective effect may be attributable to the presence of bioactive compounds in natural juices, such as vitamin C, anthocyanins, polyphenols, and, in some cases, dietary fiber (47, 48). Consistent with our findings, a U.S. cohort study also reported no significant association between natural juice consumption and EC risk (27). However, in contrast, a cohort study conducted in Canada reported a 63% increase in the risk of EC associated with high consumption of fruit juice (26). Furthermore, a comprehensive systematic review and meta-analysis on SSBs reported a 31% increase in overall cancer risk associated with the consumption of natural juices, although EC was not assessed specifically (22). Taken together, the evidence regarding the impact of natural juice consumption on EC risk remains inconclusive and may depend on the type of juice, processing methods, and the quantity consumed. Therefore, while moderate intake of natural juice may be acceptable, excessive consumption should be avoided, particularly among individuals at high risk for EC.

### 4.3 Strengths and limitations

To our knowledge, this study is among the few to concurrently evaluate the associations of beverages with EC risk. A key strength lies in the use of UK Biobank data, a large-scale prospective cohort with extensive information on diverse potential confounders. The consistency of results across multiple sensitivity and subgroup analyses further supports the robustness of our findings. Nevertheless, several limitations should be noted. First, the observational design precludes causal inference between beverage intake and EC risk. In addition, our analysis cannot verify the causal validity of the substitution findings, nor determine the duration for which replacing SSB with ASBs would need to be maintained to reduce EC risk. This important question warrants confirmation in future interventional studies. Second, beverage consumption was self-reported via 24-h dietary recalls, which are inherently susceptible to measurement error and recall bias, limiting the precision of estimating associations with absolute intake levels. Moreover, a single 24-h recall may not adequately reflect habitual dietary patterns; however, similar associations were observed in sensitivity analyses restricted to participants with more than two dietary assessments. In addition, beverage intake was collected between 2009 and 2012, whereas follow-up extended to 2022. Changes in beverage formulations, sugar-reduction initiatives, or taxation policies during this interval may have introduced non-differential exposure misclassification, potentially attenuating the true associations. Third, incident EC was identified using ICD-10 codes, which lacked detailed information to distinguish between type I and type II subtypes. Given the different etiologies of these two forms of EC (1), this limitation may have obscured subtype-specific associations. Future studies that differentiate between EC subtypes should provide more nuanced insights and contribute to the development of more targeted prevention strategies. Fourth, although the study included mediation analyses, only a partial indirect effect through BMI was observed. This suggested that BMI alone may not fully account for the complex association between beverage intake and EC risk. Future research incorporating metabolomic profiles or relevant biomarkers is therefore warranted to more comprehensively elucidate the underlying biological mechanisms. Fifth, although we adjusted for a wide range of potential confounders, residual confounding cannot be fully ruled out, which is an inherent limitation of observational studies. Notably, several dietary variables such as total energy intake, total sugar intake, and dietary quality scores contained substantial missing data and therefore could not be included in the final analyses, which may contribute to residual confounding. In addition, although non-EC mortality represents a theoretical competing event in long term cohort studies, the relatively low overall mortality and the non-lethal nature of beverage exposures suggest that competing risks are unlikely to have materially influenced the hazard ratio estimates. Nevertheless, this potential limitation should be acknowledged. Sixth, as EC develops gradually, some participants may have had undiagnosed endometrial abnormalities at baseline, introducing the potential for reverse causation. To mitigate this, we performed a sensitivity analysis excluding cases diagnosed within the first three years of follow-up. The positive association between SSBs intake and EC risk persisted, supporting the stability and robustness of our results. Seventh, as previously reported the UK Biobank is subject to healthy volunteer selection bias (49), which may have led to an underestimation of the true associations. Finally, as the UK Biobank cohort is composed mainly of middle-aged individuals of White European ancestry, the generalizability of our findings to other age groups or ethnicities may be restricted. Further research is needed to confirm these associations in more diverse populations and clarify the underlying biological mechanisms.

## 5 Conclusion

This biobank cohort study of women revealed that greater consumption of SSBs was significantly linked to elevated EC risk, while no significant associations were found for ASBs or natural juices. Substituting SSBs with other beverage types was linked to a reduced risk of EC, while reverse substitutions were associated with increased risk. These results demonstrate that choosing ASBs or natural juices might contribute to a lower risk of EC and also have potential clinical and public health relevance given the widespread consumption of SSBs. Further investigation is therefore warranted to verify these relationships and identify the biological mechanisms.

## Data Availability

The data analyzed in this study is subject to the following licenses/restrictions: access to the UK Biobank dataset is controlled and not openly available. The data is accessible to all bona fide researchers for health-related research that is in the public interest. Use of the dataset is contingent upon a formal application process, which requires approval from the UK Biobank Access Management Team. All approved research projects are governed by a signed Material Transfer Agreement (MTA), which strictly prohibits the redistribution of data to third parties and ensures that data is used solely for the approved research purposes. Requests to access these datasets should be directed to UK Biobank, https://www.ukbiobank.ac.uk/enable-your-research/apply-for-access.
