# Supplementary material for: Higher consumption of sugar-sweetened beverages is associated with an increased risk of endometrial cancer: a prospective cohort study of 92,777 women from the UK Biobank
Source: Front Public Health. 2026 Jan 7;13:1692974. doi: 10.3389/fpubh.2025.1692974 (PMC12819223; doi:10.3389/fpubh.2025.1692974)
Supplement: Supplementary file 1 [file Table_1.DOCX]

## Higher consumption of sugar-sweetened beverages is associated with an increased risk of endometrial cancer: A prospective cohort study of 92,777 women from the UK Biobank

## **Supplementary Materials Content**

Supplementary Table S1. Detailed information for the variables in this study.

Supplementary Table S2. Proportional hazards(PH) assumption test results for fully adjusted Cox models of SSBs, ASBs, and natural juices

Supplementary Table S3. Subgroup analyses for the associations between consumption of three types of beverages and the risk of endometrial cancer

Supplementary Table S4. Sensitive analysis: Risk of incident endometrial cancer by category of beverage intake after deleting the missing covariates

Supplementary Table S5. Sensitive analysis: risk of incident endometrial cancer by category of beverage intake among the participants who conducted two or more dietary assessments

Supplementary Table S6. Sensitive analysis: risk of incident endometrial cancer by category of beverage intake from the first completed dietary questionnaire

Supplementary Table S7. Sensitive analysis: risk of incident endometrial cancer by category of beverage intake, excluding participants developing events during the first 3 years of follow-up

Supplementary Table S8. Sensitive analysis: risk of incident endometrial cancer by category of beverage intake, by adjustment of household income

Supplementary Table S9. Sensitive analysis: risk of incident endometrial cancer by category of beverage intake by adjustment of household income(N=92,777)

Supplementary Table S1. Detailed information for the variables in this study.

| **Variables** | **Fields ID in the UK biobank** |
| --- | --- |
| Age | 21003 |
| Gender | 31 |
| Ethnicity | 21000 |
| Education | 6138 |
| TDI | 189 |
| Household income | 738 |
| Smoking status | 20116 |
| Drinking status | 20117 |
| Physical activity | 884, 894, 904,914 |
| HRT erver used | 2814 |
| OCs ever used | 2784 |
| Menarche age | 2714 |
| Live birth number | 2734 |
| Menopause status | 2724 |
| Ever had hysterectomy | 3591 |
| Hypertension | 93, 94, 4080, 4079, 6153, 6177 |
| Diabetes mellitus | 30750, 3074, 6153, 6177 |
| Sugar sweetened beverages | 100170, 100180 |
| Artificially sweetened beverages | 100160 |
| Natural juices | 100190, 100200, 100210 |
| Endometrial cancer | 41270, 41280, 53, 191, 40000 |

Supplementary Table S2. Proportional hazards(PH) assumption test results for fully adjusted Cox models of SSBs, ASBs, and natural juices

| **variable** | **chisq** | **df** | **p** |
| --- | --- | --- | --- |
| **Sugar beverage** | 0.6872 | 2 | 0.709 |
| age | 6.2234 | 1 | 0.013 |
| ethnicity | 0.0516 | 1 | 0.82 |
| Drinking status | 2.9295 | 2 | 0.231 |
| Smoking status | 0.6643 | 2 | 0.717 |
| TDI | 0.6705 | 1 | 0.413 |
| BMI | 0.8787 | 1 | 0.349 |
| Education | 1.8563 | 2 | 0.395 |
| Live birth number | 1.0565 | 2 | 0.59 |
| Hypertension | 4.0541 | 1 | 0.044 |
| Diabetes mellitus | 0.0536 | 1 | 0.817 |
| Menarche age | 0.198 | 1 | 0.656 |
| Menopause status | 8.6698 | 2 | 0.013 |
| HRT ever use | 0.3306 | 1 | 0.565 |
| OCs ever use | 0.8846 | 1 | 0.347 |
| Physical activity | 3.9232 | 1 | 0.048 |
|  |  |  |  |
| **Artificial beverage** | 3.0358 | 2 | 0.219 |
| age | 6.23 | 1 | 0.013 |
| ethnicity | 0.0508 | 1 | 0.822 |
| Drinking status | 2.9244 | 2 | 0.232 |
| Smoking status | 0.6656 | 2 | 0.717 |
| TDI | 0.6735 | 1 | 0.412 |
| BMI | 0.8817 | 1 | 0.348 |
| Education | 1.8349 | 2 | 0.4 |
| Live birth number | 1.0616 | 2 | 0.588 |
| Hypertension | 4.0531 | 1 | 0.044 |
| Diabetes mellitus | 0.0536 | 1 | 0.817 |
| Menarche age | 0.1993 | 1 | 0.655 |
| Menopause status | 8.662 | 2 | 0.013 |
| HRT ever use | 0.3243 | 1 | 0.569 |
| OCs ever use | 0.8909 | 1 | 0.345 |
| Physical activity | 3.9191 | 1 | 0.048 |
| GLOBAL | 27.5421 | 22 | 0.191 |
|  |  |  |  |
| **Natural juice** | 0.3424 | 2 | 0.843 |
| age | 6.221 | 1 | 0.013 |
| ethnicity | 0.0521 | 1 | 0.82 |
| Drinking status | 2.9277 | 2 | 0.231 |
| Smoking status | 0.6624 | 2 | 0.718 |
| TDI | 0.6712 | 1 | 0.413 |
| BMI | 0.8823 | 1 | 0.348 |
| Education | 1.8473 | 2 | 0.397 |
| Live birth number | 1.058 | 2 | 0.589 |
| Hypertension | 4.0509 | 1 | 0.044 |
| Diabetes mellitus | 0.0538 | 1 | 0.816 |
| Menarche age | 0.1998 | 1 | 0.655 |
| Menopause status | 8.6459 | 2 | 0.013 |
| HRT ever use | 0.3217 | 1 | 0.571 |
| OCs ever use | 0.8852 | 1 | 0.347 |
| Physical activity | 3.912 | 1 | 0.048 |
| GLOBAL | 24.9933 | 22 | 0.297 |

Abbreviations: BMI, body mass index; TDI, Townsend Deprivation Index; HRT, hormone replacement therapy; OCs, oral contraceptives; HR, hazard ratio; CI, confidence interval; ref, reference.

Model 2 adjusted for age, ethnicity, drinking status, smoking status, TDI, BMI, education, live birth number, hypertension, diabetes mellitus, menarche age, menopause status, HRT ever use, OCs ever use, and physical activity.

Supplementary Table S3. BMI-mediated proportion of the association between sugar-sweetened beverage intake and endometrial cancer risk

| Effect | HR | (95%CI) | P Value | Mediation Proportion (%) |
| --- | --- | --- | --- | --- |
| Total Effect | 1.228 | (1.113-1.355) | 0.000 |  |
| Direct Effect | 1.183 | (1.072-1.305) | 0.001 |  |
| Indirect Effect | 1.038 | (1.025-1.053) | 0.000 |  |
| Mediation Proportion |  |  |  | 18.2 |

Supplementary Table S4. Subgroup analyses for the associations between consumption of three types of beverages and the risk of endometrial cancer

|  | **Sugar-sweetened beverages (unit/day),**  **HR (95%)** | | | **Artificially sweetened beverages (unit/day),**  **HR (95%)** | | | **Natural juices (unit/day),**  **HR (95%)** | | |
| --- | --- | --- | --- | --- | --- | --- | --- | --- | --- |
| Stratification | **0** | **>0-1** | **>1** | **0** | **>0-1** | **>1** | **0** | **>0-1** | **>1** |
| Smoking status |  |  |  |  |  |  |  |  |  |
| Never | ref | 1.19(0.93,1.51) | 1.35(0.98,1.86) | ref | 0.95(0.70,1.29) | 0.96(0.66,1.37) | ref | 1.02(0.82,1.27) | 1.16(0.82,1.64) |
| Previous | ref | 1.30(0.91,1.88) | 1.43(0.87,2.33) | ref | 0.89(0.57,1.41) | 0.81(0.46,1.42) | ref | 1.35(0.98,1.85) | 1.11(0.62,2.01) |
| Current | ref | 1.45(0.57,3.69) | 1.44(0.41,5.03) | ref | 1.15(0.39,3.38) | 0.32(0.04,2.44) | ref | 1.32(0.56,3.09) | 1.76(0.49,6.29) |
| P for interaction | 0.99 | |  | 0.68 |  |  | 0.54 | |  |
| Obesity |  |  |  |  |  |  |  |  |  |
| Underweight | ref | 1.47(0.00,Inf) | 78.22(0.00,Inf) | ref | 38.04(0.00,Inf) | 0.73(0.00,Inf) | ref | 0.00(0.00,Inf) | 0.01(0.00,Inf) |
| Normal | ref | **1.43(1.01,2.02)** | 1.43(0.86,2.36) | ref | 0.88(0.52,1.50) | 1.38(0.76,2.50) | ref | 1.00(0.73,1.37) | 0.82(0.47,1.46) |
| Obese | ref | 1.09(0.77,1.53) | 1.43(0.95,2.13) | ref | 0.90(0.61,1.32) | 0.98(0.66,1.46) | ref | 1.12(0.84,1.51) | 1.28(0.79,2.07) |
| Overweight | ref | 1.20(0.85,1.69) | 1.33(0.83,2.14) | ref | 1.02(0.68,1.54) | **0.44(0.20,0.94)** | ref | 1.24(0.91,1.68) | 1.53(0.95,2.45) |
| P for interaction | 0.92 |  |  | 0.31 | |  | 0.56 | |  |
| Hypertension |  |  |  |  |  |  |  |  |  |
| No | ref | 1.28(0.87,1.87) | 1.10(0.62,1.93) | ref | 0.73(0.42,1.28) | 0.67(0.34,1.35) | ref | 0.97(0.69,1.38) | 1.24(0.72,2.13) |
| Yes | ref | 1.21(0.96,1.52) | **1.47(1.10,1.98)** | ref | 1.01(0.77,1.34) | 0.94(0.67,1.32) | ref | 1.18(0.97,1.45) | 1.16(0.82,1.63) |
| P for interaction | 0.54 | |  | 0.38 | |  | 0.61 |  |  |
| Drinking status |  |  |  |  |  |  |  |  |  |
| Never | ref | 1.96(0.94, 4.08) | 1.11(0.36, 3.42) | ref | 1.07(0.40, 2.88) | 0.76(0.22, 2.65) | ref | **3.21(1.53, 6.71)** | 1.80(0.49, 6.53) |
| Previous | ref | 1.90(0.72,5.04) | 0.41(0.05,3.24) | ref | 0.74(0.16,3.30) | 0.67(0.14,3.08) | ref | 1.31(0.48,3.54) | 1.55(0.33,7.31) |
| Current | ref | 1.16(0.94,1.43) | **1.45(1.10,1.90)** | ref | 0.94(0.72,1.22) | 0.89(0.65,1.22) | ref | 1.04(0.87,1.25) | 1.16(0.85,1.57) |
| P for interaction | 0.22 | |  | 0.98 | |  | 0.06 | |  |
| HRT ever use |  |  |  |  |  |  |  |  |  |
| No | ref | 1.19(0.93,1.53) | **1.44(1.06,1.97)** | ref | 0.92(0.67,1.25) | 0.90(0.63,1.29) | ref | 1.10(0.88,1.37) | 1.20(0.84,1.71) |
| Yes | ref | 1.30(0.94,1.80) | 1.24(0.77,2.02) | ref | 1.00(0.66,1.50) | 0.83(0.48,1.45) | ref | 1.19(0.89,1.60) | 1.17(0.70,1.94) |
| P for interaction | 0.71 | |  | 0.85 | |  | 0.85 | |  |
| OCs ever use |  |  |  |  |  |  |  |  |  |
| No | ref | 1.46(0.99,2.15) | 1.31(0.75,2.29) | ref | 0.99(0.59,1.66) | 1.20(0.66,2.19) | ref | 1.28(0.89,1.82) | 1.18(0.64,2.15) |
| Yes | ref | 1.16(0.92,1.46) | **1.40(1.04,1.88)** | ref | 0.92(0.70,1.23) | 0.80(0.57,1.14) | ref | 1.08(0.88,1.32) | 1.17(0.84,1.63) |
| P for interaction | 0.51 | |  | 0.45 | |  | 0.71 | |  |

Abbreviations: BMI, body mass index; TDI, Townsend Deprivation Index; HRT, hormone replacement therapy; OCs, oral contraceptives; HR, hazard ratio; CI, confidence interval; ref, reference.

HR was calculated based on a fully adjusted model, adjusted for age, ethnicity, drinking status, smoking status, TDI, BMI, education, live birth number, hypertension, diabetes mellitus, menarche age, menopause status, HRT ever use, OCs ever use, and physical activity. When the subgroup analyses were conducted stratified by the covariate, the covariate was not adjusted in the model. Bolded values indicate P < 0.05.

Supplementary Table S5. Sensitive analysis: Risk of incident endometrial cancer by category of beverage intake after deleting the missing covariates(N=63,750).

|  | Crude  HR (95%CI) | P | Model 1  HR (95%CI) | P | Model 2  HR (95%CI) | P |
| --- | --- | --- | --- | --- | --- | --- |
| **Sugar-sweetened beverage(unit/day)** | | | | | | |
| 0 | 1.00 (ref) |  | 1.00 (ref) |  | 1.00 (ref) |  |
| >0-1 | 1.18 (0.95-1.47) | 0.127 | 1.19 (0.96-1.48) | 0.120 | 1.13 (0.89-1.44) | 0.330 |
| >1 | 1.09 (0.8-1.48) | 0.604 | 1.12 (0.82-1.52) | 0.493 | 1.24 (0.89-1.72) | 0.197 |
| P for trend |  | 0.256 |  | 0.197 |  | 0.137 |
| **Artificial sweetened beverage(unit/day)** | | | | | | |
| 0 | 1.00 (ref) |  | 1.00 (ref) |  | 1.00 (ref) |  |
| >0-1 | 1.04 (0.8-1.36) | 0.774 | 0.96 (0.73-1.25) | 0.757 | 0.97 (0.72-1.29) | 0.815 |
| >1 | 0.9 (0.64-1.26) | 0.533 | 0.78 (0.55-1.1) | 0.150 | 0.84 (0.58-1.21) | 0.355 |
| P for trend |  | 0.671 |  | 0.175 |  | 0.377 |
| **Natural juice(unit/day)** | | | | | | |
| 0 | 1.00 (ref) |  | 1.00 (ref) |  | 1.00 (ref) |  |
| >0-1 | 0.98 (0.81-1.19) | 0.837 | 1 (0.82-1.22) | 0.977 | 1.05 (0.85-1.3) | 0.652 |
| >1 | 1.27 (0.94-1.72) | 0.114 | 1.31 (0.97-1.76) | 0.082 | 1.21 (0.86-1.7) | 0.272 |
| P for trend |  | 0.305 |  | 0.206 |  | 0.308 |

Abbreviations: BMI, body mass index; TDI, Townsend Deprivation Index; HRT, hormone replacement therapy; OCs, oral contraceptives; HR, hazard ratio; CI, confidence interval; ref, reference.

Crude model adjusted for none.

Model 1 adjusted for age, ethnicity, drinking status, smoking status, TDI, BMI, and education.

Model 2 adjusted for age, ethnicity, drinking status, smoking status, TDI, BMI, education, live birth number, hypertension, diabetes mellitus, menarche age, menopause status, HRT ever use, OCs ever use, and physical activity.

Supplementary Table S6. Sensitive analysis: risk of incident endometrial cancer by category of beverage intake among the participants who conducted two or more dietary assessments (N=56,953)

|  | Crude  HR (95%CI) | P | Model 1  HR (95%CI) | P | Model 2  HR (95%CI) | P |
| --- | --- | --- | --- | --- | --- | --- |
| **Sugar-sweetened beverage(unit/day)** | | | | | | |
| 0 | 1.00 (ref) |  | 1.00 (ref) |  | 1.00 (ref) |  |
| >0-1 | 1.23 (0.99-1.53) | 0.060 | 1.24 (0.99-1.54) | 0.057 | 1.23 (0.96-1.56) | 0.096 |
| >1 | 1.25 (0.94-1.67) | 0.132 | 1.29 (0.96-1.72) | 0.091 | 1.38 (1.01-1.88) | 0.045 |
| P for trend |  | 0.044 |  | 0.030 |  | 0.021 |
| **Artificial sweetened beverage(unit/day)** | | | | | | |
| 0 | 1.00 (ref) |  | 1.00 (ref) |  | 1.00 (ref) |  |
| >0-1 | 1.05 (0.81-1.37) | 0.696 | 0.97 (0.74-1.26) | 0.795 | 0.99 (0.74-1.33) | 0.941 |
| >1 | 0.94 (0.68-1.3) | 0.704 | 0.81 (0.58-1.13) | 0.212 | 0.9 (0.63-1.28) | 0.554 |
| P for trend |  | 0.862 |  | 0.243 |  | 0.592 |
| **Natural juice(unit/day)** | | | | | | |
| 0 | 1.00 (ref) |  | 1.00 (ref) |  | 1.00 (ref) |  |
| >0-1 | 0.99 (0.81-1.22) | 0.939 | 1.01 (0.82-1.24) | 0.912 | 1.04 (0.83-1.3) | 0.740 |
| >1 | 1.05 (0.77-1.44) | 0.750 | 1.07 (0.78-1.47) | 0.675 | 1.03 (0.72-1.46) | 0.885 |
| P for trend |  | 0.843 |  | 0.716 |  | 0.796 |

Abbreviations: BMI, body mass index; TDI, Townsend Deprivation Index; HRT, hormone replacement therapy; OCs, oral contraceptives; HR, hazard ratio; CI, confidence interval; ref, reference.

Crude model adjusted for none.

Model 1 adjusted for age, ethnicity, drinking status, smoking status, TDI, BMI, and education.

Model 2 adjusted for age, ethnicity, drinking status, smoking status, TDI, BMI, education, live birth number, hypertension, diabetes mellitus, menarche age, menopause status, HRT ever use, OCs ever use, and physical activity.

Supplementary Table S7. Sensitive analysis: risk of incident endometrial cancer by category of beverage intake from first completed dietary questionnaire(N=210,925)

|  | Crude  HR (95%CI) | P | Model 1  HR (95%CI) | P | Model 2  HR (95%CI) | P |
| --- | --- | --- | --- | --- | --- | --- |
| **Sugar-sweetened beverage(unit/day)** | | | | | | |
| 0 | 1.00 (ref) |  | 1.00 (ref) |  | 1.00 (ref) |  |
| >0-1 | 1.17 (0.84-1.62) | 0.353 | 1.2 (0.86-1.68) | 0.272 | 1.27 (0.89-1.83) | 0.191 |
| >1 | 1.17 (0.71-1.94) | 0.542 | 1.2 (0.72-1.99) | 0.489 | 1.39 (0.82-2.36) | 0.218 |
| P for trend | | 0.293 |  | 0.227 |  | 0.079 |
| **Artificial sweetened beverage(unit/day)** | | | | | | |
| 0 | 1.00 (ref) |  | 1.00 (ref) |  | 1.00 (ref) |  |
| >0-1 | 0.84 (0.52-1.35) | 0.469 | 0.78 (0.48-1.25) | 0.303 | 0.91 (0.56-1.5) | 0.721 |
| >1 | 1.02 (0.58-1.79) | 0.956 | 0.9 (0.51-1.59) | 0.724 | 1.11 (0.63-1.97) | 0.709 |
| P for trend | | 0.712 |  | 0.398 |  | 0.900 |
| **Natural juice(unit/day)** | | | | | | |
| 0 | 1.00 (ref) |  | 1.00 (ref) |  | 1.00 (ref) |  |
| >0-1 | 1.28 (1.04-1.57) | 0.021 | 1.35 (1.1-1.67) | 0.005 | 1.53 (1.23-1.91) | <0.001 |
| >1 | 1.32 (0.82-2.13) | 0.256 | 1.39 (0.86-2.25) | 0.173 | 1.32 (0.76-2.28) | 0.318 |
| P for trend | | 0.013 |  | 0.003 |  | <0.001 |

Abbreviations: BMI, body mass index; TDI, Townsend Deprivation Index; HRT, hormone replacement therapy; OCs, oral contraceptives; HR, hazard ratio; CI, confidence interval; ref, reference.

Crude model adjusted for none.

Model 1 adjusted for age, ethnicity, drinking status, smoking status, TDI, BMI, and education.

Model 2 adjusted for age, ethnicity, drinking status, smoking status, TDI, BMI, education, live birth number, hypertension, diabetes mellitus, menarche age, menopause status, HRT ever use, OCs ever use, and physical activity.

Supplementary Table S8. Sensitive analysis: risk of incident endometrial cancer by category of beverage intake, excluding participants developing events during the first 3 years of follow-up(N=92,654)

|  | Crude  HR (95%CI) | P | Model 1  HR (95%CI) | P | Model 2  HR (95%CI) | P |
| --- | --- | --- | --- | --- | --- | --- |
| **Sugar-sweetened beverage(unit/day)** | | | | | | |
| 0 | 1.00 (ref) |  | 1.00 (ref) |  | 1.00 (ref) |  |
| >0-1 | 1.22 (1-1.48) | 0.055 | 1.22 (1-1.49) | 0.048 | 1.16 (0.93-1.45) | 0.184 |
| >1 | 1.29 (0.99-1.68) | 0.063 | 1.31 (1-1.71) | 0.047 | 1.41 (1.06-1.87) | 0.018 |
| P for trend |  | 0.018 |  | 0.012 |  | 0.012 |
| **Artificial sweetened beverage(unit/day)** | | | | | | |
| 0 | 1.00 (ref) |  | 1.00 (ref) |  | 1.00 (ref) |  |
| >0-1 | 1.05 (0.83-1.34) | 0.673 | 0.96 (0.75-1.22) | 0.717 | 0.98 (0.75-1.27) | 0.853 |
| >1 | 0.89 (0.65-1.21) | 0.450 | 0.74 (0.53-1.02) | 0.066 | 0.79 (0.56-1.11) | 0.180 |
| P for trend |  | 0.629 |  | 0.087 |  | 0.223 |
| **Natural juice(unit/day)** | | | | | | |
| 0 | 1.00 (ref) |  | 1.00 (ref) |  | 1.00 (ref) |  |
| >0-1 | 1.05 (0.88-1.25) | 0.605 | 1.09 (0.91-1.3) | 0.335 | 1.13 (0.93-1.37) | 0.216 |
| >1 | 1.1 (0.82-1.48) | 0.512 | 1.14 (0.85-1.53) | 0.380 | 1.17 (0.85-1.61) | 0.329 |
| P for trend |  | 0.459 |  | 0.257 |  | 0.178 |

Abbreviations: BMI, body mass index; TDI, Townsend Deprivation Index; HRT, hormone replacement therapy; OCs, oral contraceptives; HR, hazard ratio; CI, confidence interval; ref, reference.

Crude model adjusted for none.

Model 1 adjusted for age, ethnicity, drinking status, smoking status, TDI, BMI, and education.

Model 2 adjusted for age, ethnicity, drinking status, smoking status, TDI, BMI, education, live birth number, hypertension, diabetes mellitus, menarche age, menopause status, HRT ever use, OCs ever use, and physical activity.

Supplementary Table S9. Sensitive analysis: risk of incident endometrial cancer by category of beverage intake by adjustment of household income(N=92,777)

|  | Crude  HR (95%CI) | P | Model 1  HR (95%CI) | P | Model 2  HR (95%CI) | P |
| --- | --- | --- | --- | --- | --- | --- |
| **Sugar-sweetened beverage(unit/day)** | | | | | | |
| 0 | 1.00 (ref) |  | 1.00 (ref) |  | 1.00 (ref) |  |
| >0-1 | 1.27 (1.06-1.52) | 0.008 | 1.28 (1.07-1.53) | 0.007 | 1.28 (1.07-1.53) | 0.007 |
| >1 | 1.26 (0.99-1.61) | 0.061 | 1.3 (1.01-1.66) | 0.038 | 1.29 (1.01-1.66) | 0.040 |
| P for trend | | 0.007 |  | 0.004 |  | 0.004 |
| **Artificial sweetened beverage(unit/day)** | | | | | | |
| 0 | 1.00 (ref) |  | 1.00 (ref) |  | 1.00 (ref) |  |
| >0-1 | 0.98 (0.78-1.23) | 0.875 | 0.9 (0.71-1.13) | 0.347 | 0.9 (0.71-1.12) | 0.341 |
| >1 | 0.94 (0.71-1.24) | 0.668 | 0.8 (0.6-1.06) | 0.122 | 0.79 (0.59-1.05) | 0.106 |
| P for trend | | 0.664 |  | 0.087 |  | 0.077 |
| **Natural juice(unit/day)** | | | | | | |
| 0 | 1.00 (ref) |  | 1.00 (ref) |  | 1.00 (ref) |  |
| >0-1 | 1.07 (0.91-1.25) | 0.401 | 1.11 (0.94-1.3) | 0.222 | 1.1 (0.94-1.3) | 0.230 |
| >1 | 1.21 (0.93-1.56) | 0.150 | 1.26 (0.97-1.63) | 0.078 | 1.26 (0.97-1.63) | 0.082 |
| P for trend | | 0.142 |  | 0.058 |  | 0.062 |

Abbreviations: BMI, body mass index; TDI, Townsend Deprivation Index; HRT, hormone replacement therapy; OCs, oral contraceptives; HR, hazard ratio; CI, confidence interval; ref, reference.

Crude model adjusted for none.

Model 1 adjusted for age, ethnicity, drinking status, smoking status, TDI, BMI, and education.

Model 2 adjusted for age, ethnicity, drinking status, smoking status, TDI, BMI, education, live birth number, hypertension, diabetes mellitus, menarche age, menopause status, HRT ever use, OCs ever use, and physical activity.
